# Avascular Necrosis of the Hip Compromises Gait Balance Control in Female Juveniles With Unilateral Developmental Dysplasia Treated in Toddlerhood

**DOI:** 10.3389/fbioe.2022.854818

**Published:** 2022-03-24

**Authors:** Wei-Chun Lee, Pei-An Lee, Tsan-Yang Chen, Yu-Ting Chen, Kuan-Wen Wu, Yu-Lin Tsai, Ting-Ming Wang, Tung-Wu Lu

**Affiliations:** ^1^ Department of Biomedical Engineering, National Taiwan University, Taipei, Taiwan; ^2^ Department of Orthopaedic Surgery, Chang Gung Memorial Hospital, Taipei, Taiwan; ^3^ Department of Orthopaedic Surgery, School of Medicine, National Taiwan University, Taipei, Taiwan

**Keywords:** gait, developmental dysplasia of the hip, kinematics, balance, motion analysis, avascular necrosis (AVN)

## Abstract

Avascular necrosis of the hip (AVN) is one of the most severe complications of surgical reduction when treating developmental dysplasia of the hip (DDH). The current study identified the differences in the balance control during walking in terms of the inclination angle (IA) of the center of pressure (COP) to the center of mass (COM), and the rate of change of IA (RCIA) between female juveniles with and without secondary AVN at the hip who were treated for unilateral DDH during toddlerhood as compared to their healthy peers. When compared to female healthy controls, the non-AVN group showed bilaterally similar compromised balance control with significantly decreased IA (*p* < 0.05) but increased RCIA (*p* < 0.04) in the sagittal plane during single-limb support (SLS) of the unaffected side, and in the frontal plane during terminal double-limb support (DLS) of the affected side. In contrast, the AVN increased between-side differences in the sagittal IA (*p* = 0.01), and sagittal and frontal RCIA during DLS (*p* < 0.04), leading to bilaterally asymmetrical balance control. Secondary AVN significantly reduced IA and RCIA in the sagittal plane (*p* < 0.05), and reduced range of RCIA in the frontal plane during initial DLS (*p* < 0.05). The trend reversed during terminal DLS, indicating a conservative COM-COP control in the sagittal plane and a compromised control in the frontal plane during body weight acceptance, with a compromised COM-COP control in the frontal plane during weight release. The current results suggest that increased between-side differences in the sagittal IA, and sagittal and frontal RCIA during DLS are a sign of AVN secondary to treated unilateral DDH in female juveniles, and should be monitored regularly for early identification of the disease.

## Introduction

Developmental dysplasia of the hip (DDH) is an abnormal development of the femoral head resulting in subluxation or dislocation of the hip in about 0.1–1% of the newborn population (Barlow, 1962; Gross et al., 1982; Tredwell, 1992; Storer and Skaggs, 2006). Early treatment of DDH using pelvic osteotomy has been shown to be effective in restoring hip morphology and reducing disease progression and complications such as leg length discrepancy (Michaeli et al., 1997; Nahit et al., 1998; Dwan et al., 2017; Sucato et al., 2017; Chen et al., 2018). However, residual deficits such as muscle weakness and changes of the lower limb joint mechanics remain (Storer and Skaggs, 2006), affecting the body’s balance control and dynamic stability with increased risk of falls leading to musculoskeletal injuries (Bassett GS. et al., 1999; Sturnieks et al., 2004; Pedersen et al., 2006; El-Sayed, 2009). Further, about 25–73% of children with treated DDH develop different types of avascular necrosis (AVN) of the femoral head (Kalamchi and MacEwen, 1980; Wu et al., 2012; Tang et al., 2015; Dwan et al., 2017; Sucato et al., 2017; Sankar et al., 2019). AVN secondary to treated DDH not only affects the development and biomechanics of the hip (Cooperman et al., 1983), but also predisposes the patients to premature osteoarthritis (McKay, 1974; Roach et al., 1997; Wang et al., 2016) with increased risk of falls (Lin et al., 2015).

Previous gait studies on people with treated DDH have focused mainly on the residual gait deviations (Jacobsen et al., 2006; Ömeroğlu et al., 2008; Skalshøi et al., 2015). Adolescents with treated DDH have been found to walk with reduced demands on the hip flexors and abductors, and knee extensors in the affected limb, but increased compensatory efforts from the hip extensors, ankle plantarflexors and knee flexors in the unaffected limb (Chang et al., 2012), leading to increased loading rates in both the lower limbs at heel-strike, increasing the risks of premature OA (Chang et al., 2011). No study has reported the whole-body balance control during gait and the risk of falling in patients with or without secondary AVN after pelvic osteotomy for DDH. Identifying deviations in the balance control during gait in such patients will be helpful for early detection of any fall risks and for development of preventive strategies.

The body’s dynamic balance control during gait can be evaluated in terms of the motions of the body’s center of mass (COM) in relation to the center of pressure (COP) of the ground reaction forces (GRF) (Huang et al., 2008). A common measure is the horizontal separation between the COM and COP (Hahn and Chou, 2004), although it does not consider their relative velocities, nor the height of the COM (Huang et al., 2008). These limitations have been addressed using the COM-COP inclination angle (IA) and the rate of change of IA (RCIA), which has been applied to evaluate the balance control in various subject groups (Lee and Chou, 2006; Lu et al., 2017; Lee P.-A. et al., 2021). The IA has been shown to be effective in distinguishing fallers from non-fallers in the older population (Lee and Chou, 2006) and has high test-retest reliability (De Jong et al., 2020). The IA is the angle between the COM-COP vector and the vertical, integrating the information of the horizontal COM-COP separation and the height of the COM, while the RCIA is equivalent to the angular velocity of the COM-COP if the COM-COP is considered as an inverted pendulum. In general, the greater the IA, the greater the horizontal projection of the COM deviates from the COP, and the greater the effort needed to maintain or reduce the deviation unless accompanied by an appropriate RCIA, corresponding to the position and velocity control of the COM described by Pai and Patton (Pai and Patton, 1997). In other words, an increased IA might not suggest poor balance control as long as one is able to generate an appropriate RCIA, either by changing the velocity of the COM or that of the COP, or both. The IA and RCIA together provide a more comprehensive assessment of the control of the COM relative to the COP and have been used to assess gait stability in various populations with fall risks (Pai and Patton, 1997; Hass et al., 2005; Lee and Chou, 2006; Huang et al., 2008; Said et al., 2008; Said et al., 2013; Hong et al., 2015; Lu et al., 2017; De Jong et al., 2020). While it is well known that the AVN-related increased femoral neck-shaft angle affects the resultant forces transmitted through the hip joint (Maquet, 1985), to the best knowledge of the authors, no study has reported the effects of secondary type II AVN on the whole-body balance control during gait and the risk of falling in juveniles with treated unilateral DDH by pelvic osteotomy during toddlerhood.

The purpose of the current study was to identify the alterations of the whole-body balance control during walking in terms of IA and RCIA in juveniles with treated unilateral DDH who developed secondary AVN at the treated hip and those who did not develop AVN as compared to their healthy peers. It was hypothesized that children with treated DDH, whether with or without secondary AVN, would show altered balance control with increased IA but decreased RCIA during walking when compared to female healthy controls, and that patients with AVN would show a reduced bilateral symmetry in balance control compared to those without AVN.

## Materials and Methods

### Subjects

Twenty-four female juvenile patients who had undergone Pemberton’s osteotomy for treating unilateral DDH during toddlerhood participated in the current study. Written informed assents were obtained from the participants as well as from their parents or guardians, as approved by the Institutional Review Board. The patients were divided into two groups: twelve patients in the AVN group (mean ± standard deviation of age at surgery: 2.9 ± 1.5 years, age at experiment: 7.1 ± 1.9 years, height: 119.7 ± 13.7 cm, and mass: 22.5 ± 5.5 kg) and twelve patients in the non-AVN group (non-AVN group, mean ± standard deviation of age at surgery: 2.1 ± 1.3 years, age at experiment: 7.1 ± 2.1 years, height: 123.2 ± 17.7 cm, and mass: 26.1 ± 9.5 kg) according to the osteonecrosis diagnosis and classification described by Kalamchi and MacEwen (Kalamchi and MacEwen, 1980). In the non-AVN group, ten subjects were affected on the left side and two on the right. In the AVN group, nine patients were grade II (right side: 5 and left: 4), two grade III on the left hip, and one grade IV on the right hip. The participants were community ambulatory and had not received any procedure other than Pemberton’s osteotomy. Patients with pain, infection of the hip, neuromuscular diseases or any other neurological pathology were excluded from the study. The condition of the hip at the time of experiment was quantified by center-edge angle, neck-shaft angle and acetabular index determined from the X-ray images of the pelvis in the anteroposterior view by an orthopaedic surgeon with more than 10 years of experience (WCL). The frontal-plane acetabular coverages of all the patients were all within normal range. None of the patients showed significant leg length discrepancy within 2 years follow-up after surgery, nor by the time of the gait experiment. Twelve healthy female controls (Control group, mean ± standard deviation of age: 7.6 ± 2.1 years, height: 122.1 ± 12.9 cm, and mass: 23.5 ± 4.6 kg) were also recruited to match with the non-AVN and AVN groups by sex, age, height and body weight (Table 1).

**TABLE 1:** Means (standard deviations) of radiographic parameters, demographic characteristic and gait spatiotemporal parameters in juveniles with avascular necrosis (AVN) secondary to treated unilateral DDH (AVN group, *n* = 12), without AVN (Non-AVN group, *n* = 12) and healthy controls (Control group, *n* = 12).

	AVN		*p*-value	Non-AVN		*p*-value
	Affected	Unaffected		Affected	Unaffected	
Radiographic parameters						
Acetabular index (°)	18.2 (8.2)	18.3 (6.9)	0.981	18.9 (9.3)	18.6 (4.8)	0.924
Center-edge angle (°)	16.1 (9.8)	17.5 (9.4)	0.755	25.7 (9.3)	24.7 (7.3)	0.807
Neck-shaft angle (°)	146.0 (15.8)	144.1 (9.0)	0.755	141.4 (10.2)	143.4 (9.8)	0.318
—	AVN		Non-AVN	Control		(P_A_, P_N_, P_AN_)
Demographic characteristic parameters						
Leg length (cm)	61.7 (8.5)		64.2 (12.9)	63.2 (9.9)		(0.762, 0.726, 0.769)
Age at operation (years)	2.9 (1.5)		2.1 (1.3)	—		—
Age at gait experiment (years)	7.1 (1.9)		7.1 (2.1)	7.6 (2.1)		(0.502, 0.543, 0.928)
Duration between surgery and gait experiment (years)	4.3 (1.2)		5.4 (2.2)	—		(-, -, 0.153)
Body mass (kg)	22.5 (5.5)		26.1 (9.5)	23.5 (4.6)		(0.777, 0.365, 0.243)
Body height (cm)	119.7 (13.7)		123.2 (17.7)	122.1 (12.9)		(0.464, 0.951, 0.493)
Spatiotemporal parameters						
Walking speed (mm/s)	952.1 (207.1)		1,096.6 (184.8)	1,127.1 (154.2)		(**0.026**, 0.687 0.062)
Cadence (steps/min)	124.5 (18.3)		134.5 (23.5)	141.8 (20.0)		(**0.049**, 0.395, 0.244)
Stride length (mm)	931.2 (164.9)		982.5 (79.0)	969.4 (90.3)		(0.294, 0.787, 0.432)
Step length (mm)	478.7 (50.5)		489.2 (42.4)	483.8 (45.2)		(0.789, 0.775, 0.581)
Step width (mm)	103.4 (22.7)		99.5 (16.8)	97.9 (19.0)		(0.302, 0.757, 0.302)

*p*-values for between-group comparisons: P_A_ = AVN vs. Control; P_N_ = Non-AVN vs.

Control; P_AN_ = AVN vs. Non-AVN. Bold values: significantly different.

### Experimental Protocol

In a university hospital gait laboratory, each subject walked at the preferred walking speed on a 10-m walkway while wearing 49 retro-reflective markers to track the motions of the body segments (Erdfelder et al., 1996; Chen and Lu, 2006; Chen et al., 2011). The markers were placed on anterior superior iliac spine (ASISs), posterior superior iliac spine (PSISs), greater trochanters, mid-thighs, medial and lateral epicondyles, heads of fibulae, tibial tuberosities, medial and lateral malleoli, navicular tuberosities, fifth metatarsal bases, big toes and heels, and mandibular condylar processes, acromion processes, spinal process of the seventh cervical vertebra (C7), medial and lateral humeral epicondyles, and ulnar styloids. Three-dimensional (3D) trajectories of the markers were measured at 120 Hz using an 8-camera motion capture system (Vicon MX T-40, OMG, United States), and the ground reaction forces (GRF) were measured at 1,080 Hz using three flushed forceplates (OR-6-7-1000, AMTI, United States) in the middle of the walkway. Before data collection the subjects were allowed to walk on the walkway several times. A total of six complete gait cycles were collected for each lower limb for each subject.

### Data Analysis

A 13-body-segment model was used to calculate the whole body’s COM as the mass-weighted sum of COM position vectors of all body segments defined by the measured marker data, and the forceplate data were used to calculate the COP position (Chen et al., 2011; Hsieh et al., 2011). Each body segment was embedded with a Cartesian coordinate system, with the positive *x*-axis directed anteriorly, the positive y-axis superiorly and the positive z-axis to the right. An optimization-based method, which has been shown to have better performance than traditional prediction methods (Chen et al., 2011), was used to calculate the mass and position of the COM for each body segment using the measured GRF and marker data. The COM-COP inclination angles (IA) in the sagittal and frontal planes were calculated according to the literature as follows (Hsu et al., 2010):

$$\vec{t}=\left(\vec{Z}\times \frac{{\vec{P}}_{COM-COP}}{\left|{\vec{P}}_{COM-COP}\right|}\right)$$ (1)

$$Sagittal\;IA=\sin^{-1}\left(t_{Y}\right)$$ (2)

$$Frontal\;IA=\{\begin{array}{c} -\sin^{-1}\left(t_{X}\right),\;\;\;for\;the\;right\;limb\\ \sin^{-1}\left(t_{X}\right),\;\;for\;the\;left\;limb \end{array}$$ (3)

where ${\vec{P}}_{COM-COP}$ was the vector pointing from the COP to the COM, $\vec{Z}$ was the unit vector of the vertical axis of the global coordinate system, $\vec{X}$ was the unit vector of the direction of progression, and $\vec{Y}$ was directed to the left as determined following the right-handed rule. The RCIA were calculated by smoothing and differentiating the trajectories of IA using the GCVSPL method (Woltring, 1986). A positive sagittal and frontal IA indicates that the COM is anterior to and away from the COP towards the contralateral limb, respectively. Spatiotemporal parameters were also obtained, namely walking speed, cadence, stride length, step length, and step width.

### Statistical Analysis

For statistical analysis, the sagittal and frontal IA and RCIA at heel-strike (HS) and toe-off (TO), the ranges and time-averages of the IA and RCIA during single-limb support (SLS), initial double-limb support (DLS) and terminal DLS, as well as the peak values of IA and RCIA during DLS, were obtained for each trial and for each subject. Note that initial DLS of one limb is the terminal DLS of the other limb. Data of a total of six trials were averaged for each of the variables, and the differences among non-AVN, AVN and control groups were analyzed using one-way analysis of variance (ANOVA). Once a significant main effect was found, the pair-wise differences were identified *post hoc* using independent t-tests. Between-side differences for the patient groups were tested using paired t-tests. A significance level of 0.05 was set for all tests. All statistical analyses were performed using SPSS version 20 (SPSS Inc., Chicago, IL, United States). An *a priori* power analysis using G*POWER (Erdfelder et al., 1996) based on pilot results from three participants per group determined that a projected sample size of six subjects for each group would be needed for comparisons among non-AVN, AVN and Control groups using one-way ANOVA with a power of 0.8 and a large effect size (Cohen’s f = 1.52) at a significance level of 0.05, and that a projected sample size of four subjects would be needed for comparisons between affected and unaffected sides using a paired t-test with a power of 0.8 at a significance level of 0.05 and large effect size (Cohen’s f = 3.39). Therefore, twelve subjects for each group were sufficient for the purposes of the current study. Clearance to carry out this study was provided by the Institutional Review Board.

## Results

No significant differences were found in any of the spatiotemporal parameters between the non-AVN and Control groups, nor between the AVN and non-AVN groups (Table 1). However, the AVN group showed significantly decreased walking speed and cadence when compared to Control (Table 1).

**FIGURE 1:**
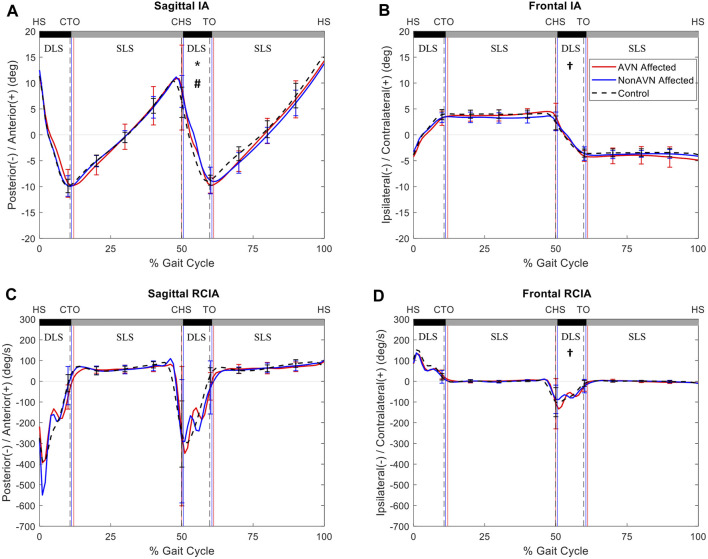
Mean curves of the COM–COP inclination angles (IA) and their rates of change (RCIA) in the sagittal **(A,C)** and frontal **(B,D)** planes for the affected side of the avascular necrosis group (AVN, red lines), non-avascular necrosis group (non-AVN, blue lines), and the control group (Control, black dashed lines) during level walking. Gait events, namely heel-strike (HS), contralateral toe-off (CTO), contralateral heel-strike (CHS) and toe-off (TO), are indicated by vertical lines. Positive sagittal and frontal IA indicate COM positions that are anterior and contralateral to the COP, respectively. Positive sagittal and frontal RCIA indicate rates of anterior changes and contralateral changes in the corresponding IA, respectively. *: significant difference between AVN and Control; †: significant difference between non-AVN and Control; #: significant difference between AVN and non-AVN.

In the sagittal plane, compared to Control, the non-AVN group showed significantly decreased average IA but significantly increased range of RCIA during SLS of the unaffected side, while the AVN group showed significantly increased average IA during terminal DLS of the affected side (i.e., initial DLS of the unaffected side). Compared to non-AVN, the AVN group showed significantly decreased RCIA at toe-off, as well as significantly decreased range of RCIA during SLS of the unaffected side, but significantly increased average IA during terminal DLS of the affected side (Tables 2, 3) (Figure 1).

**TABLE 2:** Means (standard deviations) of sagittal inclination angles (IA) for the affected and unaffected sides at heel-strike (HS) and toe-off (TO), and peak values, average values and ranges of IAs during initial double-limb support (iDLS), single-limb support (SLS) and terminal double-limb support (tDLS) in juveniles with avascular necrosis (AVN) secondary to treated unilateral DDH (AVN group, *n* = 12), without AVN (Non-AVN group, *n* = 12) and healthy controls (Control group, *n* = 12).

IA (°)						
—	AVN		Non-AVN		Control	P_AS_, P_NS_
	Affected	Unaffected	Affected	Unaffected		P_aAC_, P_aNC_, P_aAN_
						P_uAC_, P_uNC_, P_uAN_
HS	11.7 (3.5)	11.3 (2.9)	12.1 (3.3)	11.7 (2.9)	11.3 (1.7)	0.46, 0.56
						Main Effect: 0.82
						Main Effect: 0.76
TO	−10.2 (1.4)	−10.2 (2.0)	−9.7 (1.7)	−9.6 (1.2)	−9.5 (0.9)	1.00, 0.87
						Main Effect: 0.50
						Main Effect: 0.65
Peak						
iDLS	11.6 (3.3)	11.9 (2.9)	12.1 (3.3)	11.7 (2.9)	10.9 (2.0)	0.56, 0.53
						Main Effect: 0.65
						Main Effect: 0.76
tDLS	11.9 (2.9)	11.6 (3.3)	11.7 (2.9)	12.1 (3.3)	10.9 (2.0)	0.56, 0.53
						Main Effect: 0.76
						Main Effect: 0.65
Average						
iDLS	−1.9 (0.9)	−2.8 (1.2)	−1.5 (1.1)	−1.7 (1.0)	−1.9 (0.7)	**0.01**, 0.50
						Main Effect: 0.41
						**0.04**, 0.55, **0.02**
SLS	0.4 (1.5)	1.0 (1.7)	0.4 (1.0)	0.7 (0.8)	1.6 (1.3)	0.18, 0.54
						Main Effect: 0.35
						0.34, **0.04**, 0.53
tDLS	−2.8 (1.2)	−1.9 (0.9)	−1.7 (1.0)	−1.5 (1.1)	−1.9 (0.7)	**0.01**, 0.50
						**0.04**, 0.55, **0.02**
						Main Effect: 0.41
Range						
iDLS	21.9 (4.4)	21.8 (3.3)	21.8 (4.1)	21.5 (4.0)	20.5 (2.4)	0.84, 0.76
						Main Effect: 0.72
						Main Effect: 0.61
SLS	23.7 (4.3)	24.0 (5.0)	23.4 (3.7)	23.5 (3.9)	22.1 (2.4)	0.73, 0.97
						Main Effect: 0.50
						Main Effect: 0.65
tDLS	21.8 (3.3)	21.9 (4.4)	21.5 (4.0)	21.8 (4.1)	20.5 (2.4)	0.84, 0.76
						Main Effect: 0.61
						Main Effect: 0.72

Between-group caparisons for affected side: P_aNC_ = Non-AVN group vs. Control; P_aAC_ = AVN group vs. Control; P_aAN_ = AVN group vs. Non-AVN group.

Between-group caparisons for unaffected side: P_uNC_ = Non-AVN group vs. Control; P_uAC_ = AVN group vs. Control; P_uAN_ = AVN group vs. Non-AVN group.

Between-side comparisons: P_AS_ = AVN; P_NS_ = Non-AVN. Bold values: significantly different.

**TABLE 3:** Means (standard deviations) of sagittal rate of change of inclination angles (RCIA) for the affected and unaffected sides at heel-strike (HS) and toe-off (TO), and peak values, average values and ranges of IAs during initial double-limb support (iDLS), single-limb support (SLS) and terminal double-limb support (tDLS) in juveniles with avascular necrosis (AVN) secondary to treated unilateral DDH (AVN group, *n* = 12), without AVN (Non-AVN group, *n* = 12) and healthy controls (Control group, *n* = 12).

RCIA (°/s)						
—	AVN		Non-AVN		Control	P_AS_, P_NS_
	Affected	Unaffected	Affected	Unaffected		P_aAC_, P_aNC_, P_aAN_
						P_uAC_, P_uNC_, P_uAN_
HS	−179.4 (136.7)	−249.1 (194.5)	−235.4 (145.1)	−217.2 (178.0)	−244.7 (169.0)	0.22, 0.51
						Main Effect: 0.53
						Main Effect: 0.90
TO	19.9 (31.9)	−16.1 (33.2)	−45.6 (103.5)	−40.3 (64.1)	−31.9 (75.2)	**0.02**, 0.88
						0.61, 0.08, **0.05**
						Main Effect: 0.61
Peak						
iDLS	−524.7 (221.3)	−595.9 (239.6)	−658.6 (303.1)	−621.3 (223.9)	−370.9 (124.8)	**0.03**, 0.38
						Main Effect: 0.14
						Main Effect: 0.16
tDLS	−595.9 (239.6)	−524.7 (221.3)	−621.3 (223.9)	−658.6 (303.1)	−370.9 (124.8)	**0.03**, 0.38
						Main Effect: 0.16
						Main Effect: 0.14
Average						
iDLS	−205.6 (79.1)	−199.6 (65.2)	−258.8 (127.0)	−248.0 (85.3)	−242.1 (68.5)	0.66, 0.59
						Main Effect: 0.34
						Main Effect: 0.23
SLS	57.9 (12.4)	61.8 (15.4)	63.6 (16.3)	62.8 (16.9)	58.4 (13.3)	0.20, 0.60
						Main Effect: 0.55
						Main Effect: 0.40
tDLS	−199.6 (65.2)	−205.6 (79.1)	−248.0 (85.3)	−258.8 (127.0)	−242.1 (68.5)	0.66, 0.59
						Main Effect: 0.23
						Main Effect: 0.34
Range						
iDLS	519.1 (205.6)	631.1 (243.2)	658.0 (289.4)	615.9 (207.2)	617.8 (124.8)	**< 0.01**, 0.30
						Main Effect: 0.28
						Main Effect: 0.98
SLS	287.5 (258.4)	128.2 (74.4)	249.9 (104.2)	280.2 (176.6)	106.6 (50.5)	0.08, 0.57
						Main Effect: 0.38
						0.41, **0.01**, **0.02**
tDLS	631.1 (243.2)	519.1 (205.6)	615.9 (207.2)	658.0 (289.4)	617.8 (124.8)	< **0.01**, 0.30
						Main Effect: 0.98
						Main Effect: 0.28

Between-group caparisons for affected side: P_aNC_ = Non-AVN group vs. Control; P_aAC_ = AVN group vs. Control; P_aAN_ = AVN group vs. Non-AVN group.

Between-group caparisons for unaffected side: P_uNC_ = Non-AVN group vs. Control; P_uAC_ = AVN group vs. Control; P_uAN_ = AVN group vs. Non-AVN group.

Between-side comparisons: P_AS_ = AVN; P_NS_ = Non-AVN. Bold values: significantly different.

**FIGURE 2:**
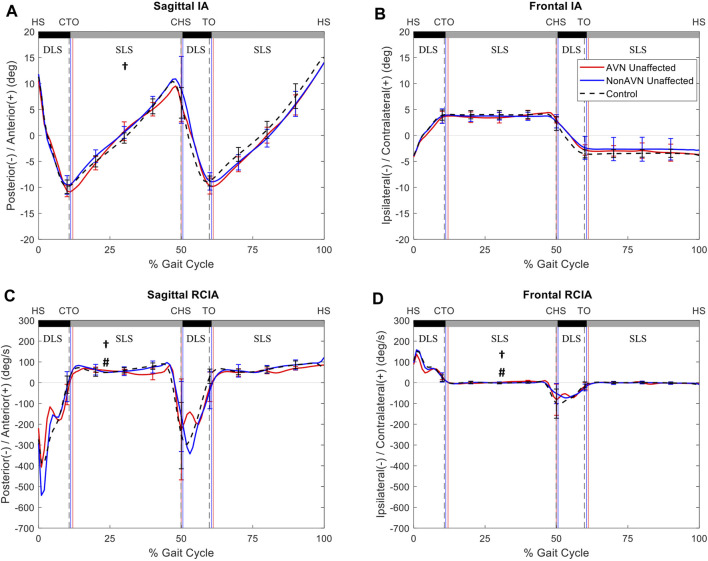
Mean curves of the COM–COP inclination angles (IA) and their rates of change (RCIA) in the sagittal **(A,C)** and frontal **(B,D)** planes for the unaffected side of the avascular necrosis group (AVN, red lines), non-avascular necrosis group (non-AVN, blue lines), and the control group (Control, black dashed lines) during level walking. Gait events, namely heel-strike (HS), contralateral toe-off (CTO), contralateral heel-strike (CHS) and toe-off (TO), are indicated by vertical lines. Positive sagittal and frontal IA indicate COM positions that are anterior and contralateral to the COP, respectively. Positive sagittal and frontal RCIA indicate rates of anterior changes and contralateral changes in the corresponding IA, respectively. *: significant difference between AVN and Control; †: significant difference between non-AVN and Control; #: significant difference between AVN and non-AVN.

In the frontal plane, compared to Control, the non-AVN group showed significantly decreased average IA but significantly increased peak value of RCIA during terminal DLS of the affected side, while the AVN group showed significantly decreased RCIA at toe-off but increased range of RCIA during SLS of the unaffected side. Compared to non-AVN, the AVN group showed significantly decreased RCIA at toe-off and significantly decreased range of RCIA during SLS of the unaffected side (Tables 4, 5) (Figure 2).

**TABLE 4:** Means (standard deviations) of frontal inclination angles (IA) for the affected and unaffected sides at heel-strike (HS) and toe-off (TO), and peak values, average values and ranges of IAs during initial double-limb support (iDLS), single-limb support (SLS) and terminal double-limb support (tDLS) in juveniles with avascular necrosis (AVN) secondary to treated unilateral DDH (AVN group, *n* = 12), without AVN (Non-AVN group, *n* = 12) and healthy controls (Control group, *n* = 12).

IA (°)						
—	AVN		Non-AVN		Control	P_AS_, P_NS_
	Affected	Unaffected	Affected	Unaffected		P_aAC_, P_aNC_, P_aAN_
						P_uAC_, P_uNC_, P_uAN_
HS	−4.2 (1.5)	−3.9 (1.5)	−3.5 (0.9)	−3.9 (1.1)	−3.9 (1.5)	0.26, 0.33
						Main Effect: 0.44
						Main Effect: 0.99
TO	−3.9 (1.3)	−3.3 (1.5)	−3.7 (1.1)	−2.9 (1.8)	−3.5 (0.8)	0.13, 0.28
						Main Effect: 0.65
						Main Effect: 0.33
Peak						
iDLS	4.4 (1.8)	3.8 (1.7)	3.5 (0.9)	3.9 (1.1)	3.9 (0.9)	0.09, 0.33
						Main Effect: 0.33
						Main Effect: 0.99
tDLS	3.8 (1.7)	4.4 (1.8)	3.9 (1.1)	3.5 (0.9)	3.9 (0.9)	0.09, 0.33
						Main Effect: 0.99
						Main Effect: 0.33
Average						
iDLS	0.3 (0.9)	−0.4 (1.3)	−0.5 (1.2)	−0.1 (0.6)	−0.6 (0.6)	0.22, 0.19
						Main Effect: 0.52
						0.69, **0.05**, 0.42
SLS	3.5 (1.6)	3.8 (1.7)	3.0 (2.0)	3.8 (0.8)	4.0 (0.8)	0.52, 0.28
						Main Effect: 0.28
						Main Effect: 0.76
tDLS	−0.4 (1.3)	0.3 (0.9)	−0.1 (0.6)	−0.5 (1.2)	−0.6 (0.6)	0.22, 0.19
						0.69, **0.05**, 0.42
						Main Effect: 0.52
Range						
iDLS	8.0 (2.0)	8.3 (1.4)	6.8 (1.3)	7.7 (1.7)	7.3 (1.4)	0.58, 0.15
						Main Effect: 0.29
						Main Effect: 0.24
SLS	1.6 (0.9)	1.6 (0.8)	1.4 (0.5)	1.3 (0.3)	1.2 (0.5)	0.94, 0.44
						Main Effect: 0.36
						**0.02**, 0.06, 0.15
tDLS	8.3 (1.4)	8.0 (2.0)	7.7 (1.7)	6.8 (1.3)	7.3 (1.4)	0.58, 0.15
						Main Effect: 0.24
						Main Effect: 0.29

Between-group caparisons for affected side: P_aNC_ = Non-AVN group vs. Control; P_aAC_ = AVN group vs. Control; P_aAN_ = AVN group vs. Non-AVN group.

Between-group caparisons for unaffected side: P_uNC_ = Non-AVN group vs. Control; P_uAC_ = AVN group vs. Control; P_uAN_ = AVN group vs. Non-AVN group.

Between-side comparisons: P_AS_ = AVN; P_NS_ = Non-AVN. Bold values: significantly different.

**TABLE 5:** Means (standard deviations) of frontal rate of change of inclination angles (RCIA) for the affected and unaffected sides at heel-strike (HS) and toe-off (TO), and peak values, average values and ranges of IAs during initial double-limb support (iDLS), single-limb support (SLS) and terminal double-limb support (tDLS) in juveniles with avascular necrosis (AVN) secondary to treated unilateral DDH (AVN group, *n* = 12), without AVN (Non-AVN group, *n* = 12) and healthy controls (Control group, *n* = 12).

RCIA (°/s)						
—	AVN		Non-AVN		Control	P_AS_, P_NS_
	Affected	Unaffected	Affected	Unaffected		P_aAC_, P_aNC_, P_aAN_
						P_uAC_, P_uNC_, P_uAN_
HS	69.2 (50.5)	99.6 (68.9)	70.0 (55.1)	79.1 (44.2)	92.2 (49.2)	0.12, 0.80
						Main Effect: 0.47
						Main Effect: 0.62
TO	11.0 (8.3)	18.1 (6.1)	−28.3 (31.9)	−20.2 (12.3)	−17.0 (11.4)	**0.01**, 0.47
						0.06, 0.26, **0.04**
						Main Effect: 0.40
Peak						
iDLS	−161.4 (88.4)	−194.9 (96.2)	−162.7 (69.1)	−189.2 (39.1)	−133.4 (68.9)	0.07, 0.26
						Main Effect: 0.91
						0.09, **0.02**, 0.85
tDLS	−194.9 (96.2)	−161.4 (88.4)	−189.2 (39.1)	−162.7 (69.1)	−133.4 (68.9)	0.07, 0.26
						0.09, **0.02**, 0.85
						Main Effect: 0.91
Average						
iDLS	−70.5 (31.1)	−74.2 (30.9)	−68.4 (35.8)	−84.6 (18.8)	−85.6 (21.3)	0.52, 0.15
						Main Effect: 0.21
						Main Effect: 0.45
SLS	−0.2 (2.8)	−0.1 (4.4)	−1.3 (2.0)	−0.6 (2.4)	−0.0 (2.2)	0.96, 0.50
						Main Effect: 0.38
						Main Effect: 0.75
tDLS	−74.2 (30.9)	−70.5 (31.1)	−84.6 (18.8)	−68.4 (35.8)	−85.6 (21.3)	0.52, 0.15
						Main Effect: 0.45
						Main Effect: 0.21
Range						
iDLS	152.4 (74.3)	200.2 (77.9)	158.0 (52.9)	173.6 (33.0)	175.7 (45.4)	**0.01**, 0.38
						Main Effect: 0.43
						Main Effect: 0.44
SLS	98.5 (88.4)	37.0 (23.0)	66.8 (36.1)	75.8 (56.7)	119.2 (70.9)	0.06, 0.66
						0.53, **0.03**, 0.27
						0.92, 0.08, **0.04**
tDLS	200.2 (77.9)	152.4 (74.3)	173.6 (33.0)	158.0 (52.9)	175.7 (45.4)	**0.01**, 0.38
						Main Effect: 0.44
						Main Effect: 0.43

Between-group caparisons for affected side: P_aNC_ = Non-AVN group vs. Control; P_aAC_ = AVN group vs. Control; P_aAN_ = AVN group vs. Non-AVN group.

Between-group caparisons for unaffected side: P_uNC_ = Non-AVN group vs. Control; P_uAC_ = AVN group vs. Control; P_uAN_ = AVN group vs. Non-AVN group.

Between-side comparisons: P_AS_ = AVN; P_NS_ = Non-AVN. Bold values: significantly different.

**FIGURE 3:**
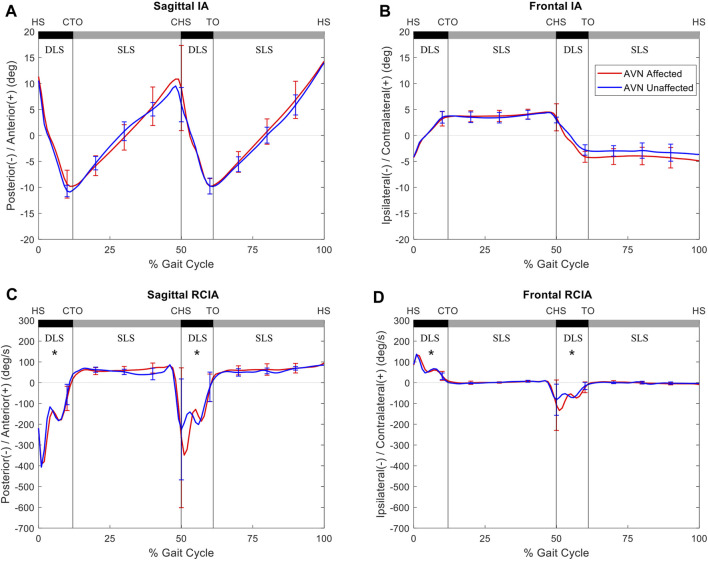
Mean curves of the COM–COP inclination angles (IA) and their rates of change (RCIA) in the sagittal **(A,C)** and frontal **(B,D)** planes for the avascular necrosis group of the affected side (red lines) and unaffected side (blue lines) during level walking. Gait events, namely heel-strike (HS), contralateral toe-off (CTO), contralateral heel-strike (CHS) and toe-off (TO), are indicated by vertical lines. Positive sagittal and frontal IA indicate COM positions that are anterior and contralateral to the COP, respectively. Positive sagittal and frontal RCIA indicate rates of anterior changes and contralateral changes in the corresponding IA, respectively. *: significant difference between sides.

**FIGURE 4:**
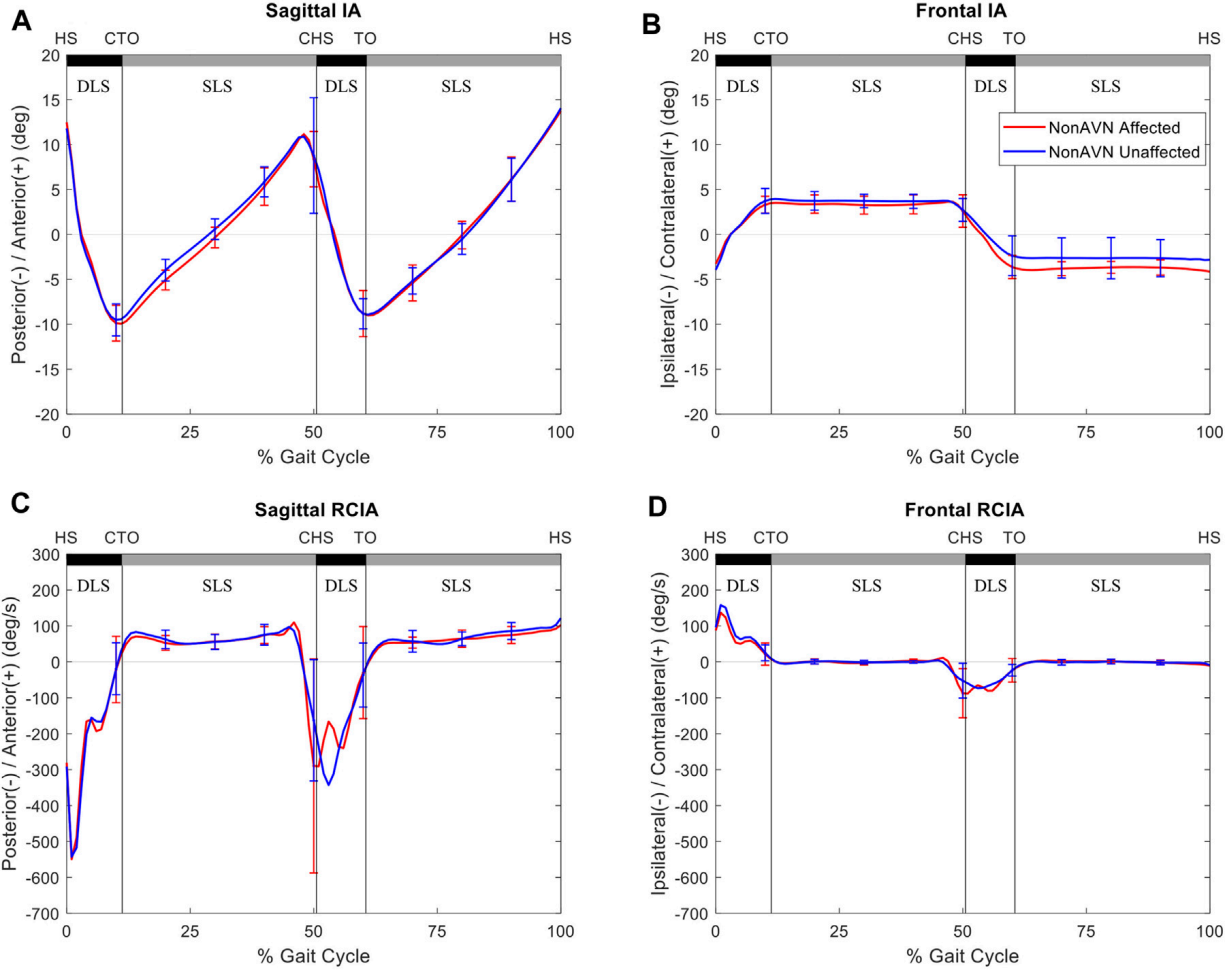
Mean curves of the COM–COP inclination angles (IA) and their rates of change (RCIA) in the sagittal **(A,C)** and frontal **(B,D)** planes for the non-avascular necrosis group of the affected side (red lines) and unaffected side (blue lines) during level walking. Gait events, namely heel-strike (HS), contralateral toe-off (CTO), contralateral heel-strike (CHS) and toe-off (TO), are indicated by vertical lines. Positive sagittal and frontal IA indicate COM positions that are anterior and contralateral to the COP, respectively. Positive sagittal and frontal RCIA indicate rates of anterior changes and contralateral changes in the corresponding IA, respectively.

Compared to the unaffected side, the affected side of the AVN group showed significantly decreased average IA, as well as significantly decreased RCIA at toe-off, peak value of RCIA and range of RCIA during initial DLS in the sagittal plane, but significantly increased RCIA at toe-off and significantly decreased range of RCIA during initial DLS in the frontal plane (Figure 3). No significant differences were found in the IA- and RCIA-related variables between the affected and unaffected sides in the non-AVN group (Figure 4).

## Discussion

The current study aimed to identify the alterations of the whole-body balance control during walking in terms of IA and RCIA of COM relative to COP in juveniles who developed secondary AVN at the hip after treatment for DDH during toddlerhood and those without developing AVN as compared to their healthy peers. When compared to female healthy controls, the non-AVN group showed bilaterally similar compromised balance control with significantly decreased IA but increased RCIA in the sagittal plane during SLS of the unaffected side, and in the frontal plane during terminal DLS of the affected side (i.e., initial DLS of the unaffected side). In contrast, the secondary AVN increased between-side differences in the sagittal IA and sagittal and frontal RCIA during DLS, leading to altered, bilaterally different balance control in the AVN group. This group showed significantly increased IA but decreased RCIA in the sagittal plane during terminal DLS of the affected side and in the frontal plane during SLS of the unaffected side compared to female healthy controls. Comparisons between the AVN and non-AVN groups showed that AVN secondary to unilateral DDH treatment increased sagittal IA during terminal DLS of the affected side and reduced both the sagittal and frontal RCIA during SLS of the unaffected side. The current results suggest that apart from routine assessment of the morphological changes of the affected hip, it is essential to monitor for any signs of decreased gait speed and cadence or increased between-side differences in the sagittal IA, and sagittal and frontal RCIA during DLS for early identification of AVN subsequent to unilateral DDH osteotomy in juveniles.

Juveniles without secondary AVN walked with compromised, bilaterally similar balance control showing reduced IA and increased RCIA in the sagittal plane during SLS of the unaffected side, and in the frontal plane during terminal DLS of the affected side at normal gait speed, cadence, stride length, step length and step width. When supported only by the unaffected limb, the COM moved anteriorly at an increased speed while the COP was controlled within a relatively small range, suggesting a compromised velocity control of the COM-COP motion in the sagittal plane. During DLS, on the other hand, the COP moved towards the leading limb during weight transfer; thus, an increased RCIA during terminal DLS of the affected limb indicated a faster weight release to the unaffected side. These changes of the COM-COP control appeared to be a compensation for residual deficits of the hip such as muscle weakness, limited range of motion and modified mechanical properties of the surgically reduced joint (Rab, 1978; Bassett G. S. et al., 1999; Tavares, 2004; Lovell et al., 2006; Pedersen et al., 2006; Wu et al., 2020). Reduced muscle strengths have been shown to affect balance control (Storer and Skaggs, 2006). The release of psoas and adductor longus muscles, and altered muscle lines of action as a result of the realignment of the bony structures during the osteotomy might also contribute to the observed changes in balanced control. Nonetheless, given the unilateral residual deficits, the current subjects were able to achieve a more symmetrical bilateral control without significant between-side differences in the COM-COP control a few years after the surgery, presumably *via* compensatory changes.

The development of AVN secondary to the unilateral DDH treatment degraded or destroyed the hip biomechanics reconstructed by the osteotomy, increasing the difficulties in maintaining balance for the affected limb and in providing compensatory adjustments by the unaffected limb necessary for symmetrical balance control. In the sagittal plane, the juveniles with secondary AVN walked with increased IA but reduced RCIA during terminal DLS of the affected limb, indicating a compromised COM-COP control during weight release to the leading unaffected limb at a reduced speed. A plausible reason for this phenomenon is that AVN of the femoral head affects the biomechanics of the hip joint and the surrounding muscles, reducing the unresisted range of motion of the joint and the ability for generating power necessary for body-weight transfer from the trailing limb (affected side) to the leading limb (unaffected side). Reduced speed in weight transfer to the leading limb as a result of reduced forward propulsion of the affected trailing limb during terminal DLS further affects the balance control with reduced frontal RCIA during the subsequent SLS of the unaffected side.

In the frontal plane, the patients with AVN also showed a compromised COM-COP control with increased IA but reduced RCIA during SLS of the unaffected limb. During this period the control of the COM motion relative to the COP vector can be considered as an inverted pendulum with the COP controlled within a small range. However, the COM moved beyond the unaffected foot while the affected limb swung from the trailing to the leading position. With the increased IA an increase in the corresponding RCIA would be needed for stable balance control (Pai and Patton, 1997). The patients with AVN did not show the necessary increase in the RCIA. Instead, the reduced RCIA is most likely the result of the reduced ability of velocity control of the affected limb, indicating a less stable COM-COP control with an increased risk of loss of balance.

The non-AVN group walked with similar COM-COP balance control strategies between affected and unaffected side, while those for the AVN group were bilaterally different mainly in the sagittal IA, and sagittal and frontal RCIA during DLS. Secondary AVN significantly reduced IA and RCIA in the sagittal plane, and reduced the range of RCIA in the frontal plane during initial DLS, with a reversed trend during terminal DLS as the trailing limb became the leading limb. These results indicate a conservative COM-COP control in the sagittal plane and a compromised control in the frontal plane during body-weight transfer to the leading affected limb, and a compromised COM-COP control in the frontal plane during weight release to the unaffected limb. These strategies were most likely related to the altered biomechanical conditions of the affected hip, including the changes in the shape and orientation of the femoral head and the neck-shaft angle, as well as the lines of action and lever-arm lengths of the surrounding muscles (Rab, 1978; Bassett GS. et al., 1999; Tavares, 2004; Lovell et al., 2006; Wu et al., 2020). Therefore, increased between-side differences in the sagittal IA, and sagittal and frontal RCIA during DLS of level walking appeared to be a sign of AVN secondary to unilateral DDH osteotomy in juveniles, and these differences may be used for early diagnosis of AVN in children with treated unilateral DDH.

The current study was the first to identify the effects of AVN secondary to surgically treated unilateral DDH on the whole-body balance control and bilateral asymmetry of such control during walking in terms of IA and RCIA in juveniles. The bilateral asymmetry as a result of secondary AVN was found to occur mainly in the sagittal IA, and sagittal and frontal RCIA during DLS, while the COM-COP control remained symmetrical during SLS. A recent study showed that the loading rates on both the affected and unaffected sides were strongly correlated to the morphology of both the affected and unaffected hips (Lee W.-C. et al., 2021). Therefore, further study on the possible relationship between hip morphology parameters and IA and RCIA may help identify the possible mechanisms for the observed balance deviations in secondary AVN in the current patient population. The current subjects with AVN were limited to female patients with unilateral DDH, reflecting the 5–9 times higher prevalence of DDH in females than in males (Dunn et al., 1985). For males and those with bilateral DDH, further studies will be needed to evaluate whether COM-COP control strategies similar to the current patient population would be adopted. Further longitudinal gait studies are also needed to identify the effects of the severity of AVN secondary to pelvic osteotomy on the COM-COP control during walking. The current subjects were evaluated at their preferred walking speeds, reflecting their physical and control abilities. Further studies on the subjects walking slower and faster than the preferred speeds may be needed to reveal the effects of gait speed on the balance control. It is noted that the current study focused on the balance control during unobstructed level walking, the fundamental activity of human locomotion. Complete knowledge of level walking is clinically important and can also provide baseline data for further studies on more challenge activities including obstacle-crossing.

## Conclusion

Without secondary AVN, female juveniles who were treated for unilateral DDH during toddlerhood walked with bilaterally similar compromised balance control, showing significantly decreased IA but increased RCIA in the sagittal plane during SLS of the unaffected side, and in the frontal plane during terminal DLS of the affected side. Secondary AVN significantly reduced IA and RCIA in the sagittal plane, and reduced range of RCIA in the frontal plane during initial DLS, with a reversal during terminal DLS, indicating a conservative COM-COP control in the sagittal plane and a compromised control in the frontal plane during body-weight acceptance, and a compromised COM-COP control in the frontal plane during weight release. These strategies were most likely related to the altered biomechanical conditions of the affected hip, including the changes in the shape and orientation of the femoral head and the neck-shaft angle, as well as the lines of action and lever-arm lengths of the surrounding muscles. The current results suggest that increased between-side differences in the sagittal IA, and sagittal and frontal RCIA during DLS of level walking are a sign of AVN secondary to unilateral DDH osteotomy in female juveniles. These differences should be monitored for early identification of AVN secondary to treated unilateral DDH in this patient population.

## Data Availability

The original contributions presented in the study are included in the article/Supplementary Material, further inquiries can be directed to the corresponding authors.
